# High prevalence of *bla*_CTX-M_ and *bla*_SHV_ among ESBL producing *E. coli* isolates from beef cattle in China’s Sichuan-Chongqing Circle

**DOI:** 10.1038/s41598-021-93201-z

**Published:** 2021-07-02

**Authors:** Yu-Long Zhang, Fang-Yuan Huang, Lin-Li Gan, Xin Yu, Dong-Jie Cai, Jing Fang, Zhi-jun Zhong, Hong-rui Guo, Yue Xie, Jun Yi, Zhi-sheng Wang, Zhi-Cai Zuo

**Affiliations:** 1 Key Laboratory of Animal Disease and Human Health of Sichuan Province, College of Veterinary Medicine, Sichuan Agricultural University, Sichuan Province, Ya’an, 625014 China; 2 Sichuan Academy of Agricultural Sciences, Sichuan Province, Chengdu, 610066 China; 3 Animal Nutrition Institute, Sichuan Agricultural University, Chengdu, Sichuan 611130 China

**Keywords:** Microbiology, Molecular biology

## Abstract

Enterobacteria that produce extended-spectrum *β-lactamase* (ESBL) such as *Escherichia coli *(*E. coli*) are common in our environment and known to cause serious health implications in humans and animals. *β-lactam* antibiotics such as penicillins, cephalosporins and monobactams are the most commonly used anti-bacterials in both humans and animals, however, Gram negative bacteria (such as *E. coli*) that produces extended-spectrum *β-lactamases* (ESBLs) have the ability to hydrolyze most *β-lactams* therefore making them resistant to *β-lactam* antibiotics. Recent extensive researches on the epidemiology and genetic characteristics of extended-spectrum β-lactamase (ESBL)-producing *E. coli* reported the existence of ESBL-producing *E. coli* in humans, companion animals and poultry. Therefore, this experiment was performed to investigate the prevalence and genetic characteristics of *β-lactamase* producing *E. coli* isolated from beef cattle farms in the Sichuan-Chongqing circle of China. Phenotypic confirmation of ESBL-producing *E. coli w*as performed using the double disk synergy test. Polymerase Chain Reaction (PCR) was used to detect *bla*_CTX-M_, *bla*_SHV_ and *bla*_TEM_ gene codes, then after, isolates were divided into different phylogenetic groups and multi-locus sequence typing (MLST). The results showed that out of the 222 *E. coli* strains isolated from the beef cattle, 102 strains showed ESBL phenotypes. The PCR results showed that *bla*_CTX-M_ was the predominant ESBL gene identified among the *E. coli* strains with 21 (9.5%) isolates having this gene, followed by *bla*_SHV_ which was found in 18 (8.1%) isolates. The majority of these ESBL positive isolates were assigned to phylogroup A (19.8%) followed by phylogroup B1 (13.5%). In addition, from the MLST results on ESBL positive isolates (n = 30) we identified 19 STs, ST398 (ST398cplx) and ST7130 which were the prevalent population (20%). In conclusion, the high prevalence of CTX-M, and SHV in the study confirmed its association with *E. coli* infection; therefore, this calls for health concerns on ESBL-producing *E. coli*. As far as we know, this is the first comprehensive research report relating to ESBL-producing *E. coli* incidence in Chinese beef cattle.

## Introduction

*Escherichia coli* is one of the important bacteria species that cause several bacterial infections both in humans and animals^1^. Pathogenic *E. coli* can cause a wide variety of intestinal and extraintestinal infections, such as urinary tract infections (UTIs), neonatal meningitis, sepsis, skin and soft tissue infections (SSTIs), and colisepticemia^2,3^. *Escherichia coli* can survive and adapt in a variety of external environments, and spread between humans, animals and their products as well as various channels, such as direct contact with humans, animals or eating contaminated food^4^. From a global perspective, the treatment of bacterial diseases still require the use of antibiotics, but antibiotic resistance due to irrational use has now become a serious public health problem^5,6^. Extended-spectrum *β-lactamases* (ESBLs) are enzymes that can hydrolyze a large variety of *β-lactam* antibiotics, including penicillins, cephalosporins and monobactams, making gram-negative bacteria such as *E. coli* resistant to *β-lactam* antibiotics^7^. The emergence of antibiotic resistance in bacteria have affected the effectiveness of antibiotics in treating bacterial infections. The increasing prevalence of multidrug-resistant *E. coli* strains worldwide as a result of hydrolase production and spread of mobile genetic elements such as broad-spectrum multidrug-resistant lactamase (ESBLs) by *E. coli*, poses a clinical and epidemiological challenge^1,8^.

Some of the most deleterious enzymes in the clinical practice, such as extended-spectrum *β-lactamases* (ESBLs) and metallo-carbapenemases, are partially related to the new *β-lactamase* inhibitor combinations^9,10^. The occurrence and dissemination of ESBL-producing *E. coli* is ubiquitous in farms and the environment^11,12^. Food-producing animals, including poultry, pigs and cattle are potentially at risk to the dissemination of ESBL-producing *E. coli*^13–15^. The high and increasing occurrence of ESBL-producing *E. coli* in the environment has drawn the attention of a slew of researchers. In Enterobacteriaceae, the dramatic increase in the rates of resistance to penicillins, cephalosporins and monobactams mainly results from the spread of plasmid-borne extended-spectrum *β-lactamase* (ESBL), especially the plasmids containing the CTX-M family^16^. The CTX-M-type extended-spectrum *β-lactamase* (ESBL) gene *bla*_CTX-M_ is mainly carried by antimicrobial resistance plasmids. Currently more than 150 allelic variants of CTX-M have been identified, and the continuous increase of these variants are responsible for the serious therapeutic issues^17,18^. Among all the clinical *E. coli* isolation across the world (especially China), CTX-M is widely distributed and is the major genotype of ESBL^19–21^.

An important public health and clinical concern is that, pandemicity itself may be a determinant of progressive drug resistance acquisition by the clonal lineages^22^. Clonal relationship reported that there is an existing knowledge gap between the resistance determinants and multilocus sequencing typing (MLST) when it comes to the geographical distribution of isolates^23,24^. However, studies from Europe and North America showed similar distributions of ExPEC STs, but those from Asian and African did not. Persistence, adaptation, and predominance in the intestinal reservoir may drive ExPEC success^25^.

Certain international high-risk multidrug-resistant clones such as ST10 isolated from pigs^26,27^, ST410 from urinary tract^28^ and ST398 have been identified^29,30^. *E. coli* clonal group A constitutes an important clonal lineage among extraintestinal pathogenic *E. coli*, because they cause serious disease such as urinary tract infections (UTI), and bacteraemia in humans^31^.

Extended-spectrum *β-lactamases* (ESBLs) are a group of enzymes that can hydrolyze a variety of *β-lactams*, including the fourth-generation cephalosporins, and compromise all the effects of *β-lactams*, except cephamycins and carbapenems^32^. The emergence of extended-spectrum *β-lactamase* species has caused serious infections and diseases in human and livestock worldwide^32,33^. However, studies on the ESBL-producing *E. coli* isolated from beef cattle are limited, therefore, in this current study, the prevalence and molecular characteristics of *E. coli* harboring ESBL genes was investigated in 222 isolates of nonduplicate cloacal swabs obtained from 15 beef cattle farms, to provide the inclusive and consistent epidemiological information to aid in developing preventive strategies for the rapid propagation of ESBL in animal products such as beef in the Sichuan-Chongqing Circle of China.

## Methods

### Study location

**Figure 1:**
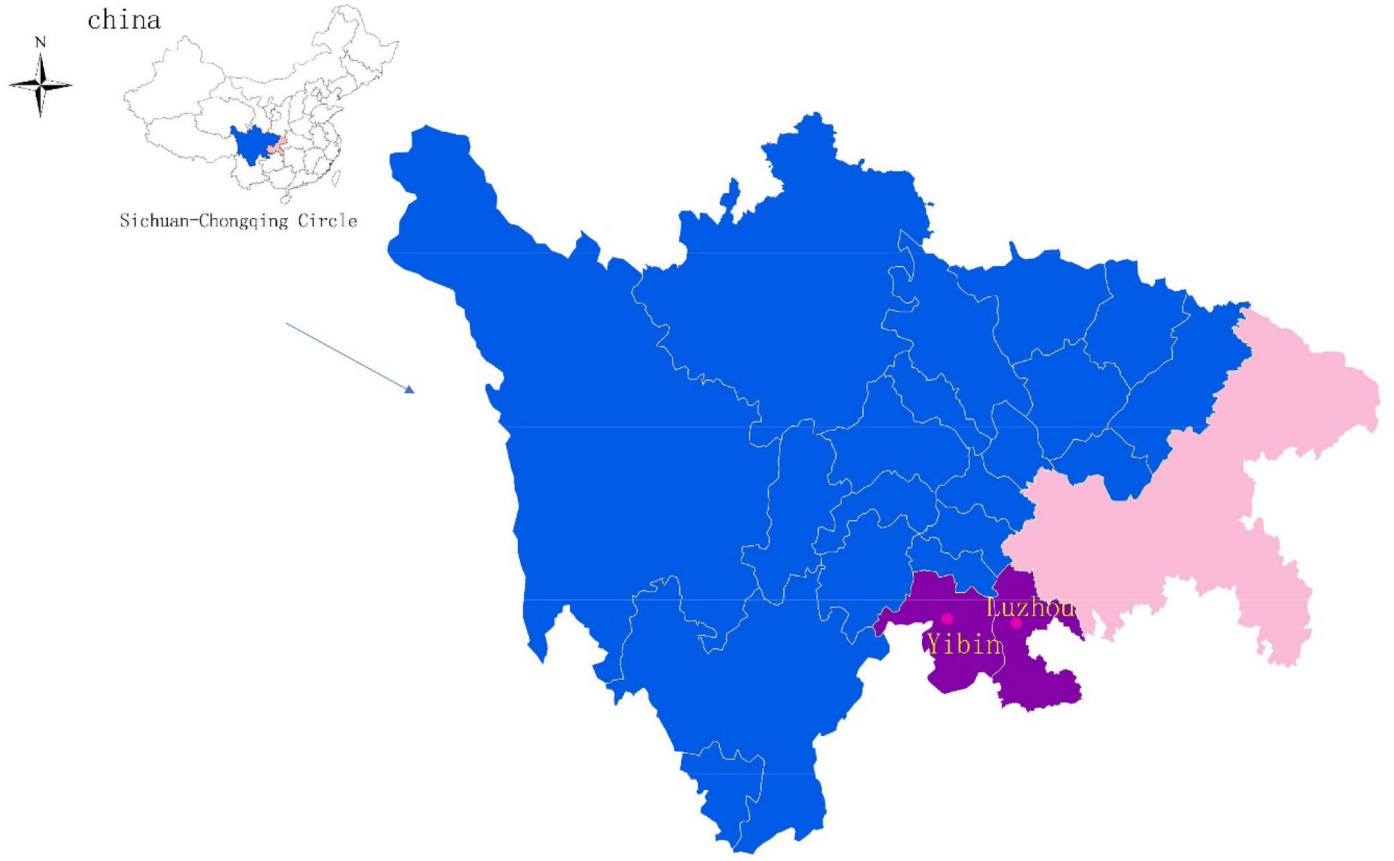
A map created by Mapinfo software (Version 17.0.2, Database: OnlineMap), showing the sampling sites in the Sichuan-Chongqing Circle of China. All the samples were collected from 15 different farms in Yibin and Luzhou districts.

The Sichuan-Chongqing Circle covers an area of 0.21 million square kilometers, accounting for approximately 2.1% of mainland China (Fig. 1). Two cities from this region Yibin City and Luzhou City are noted for their well-developed and large-scale beef industries. According to an unpublished survey report in 2019, the beef cattle industries in Yibin and Luzhou were estimated to be worth 1.6 billion dollars, while the total numbers of beef cattle were 1 million.

The Sichuan-Chongqing Circle has huge geographical advantages and is a strategic fulcrum for the development of the western region and a connection point for China's "One Belt, One Road" strategy. The Sichuan-Chongqing circle fully cooperate with the Yangtze River Delta circle to promote the coordinated development of regional economy and the development of the southwest region.

### Sample collection, isolation and identification of *E. coli*

**Figure 2:**
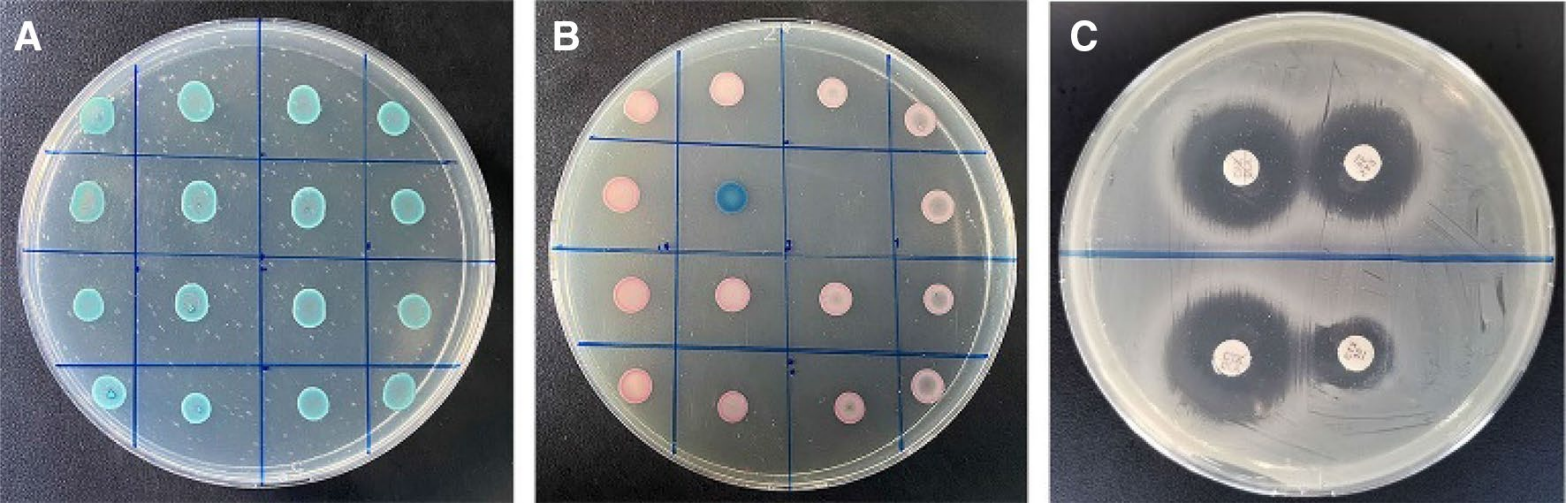
(**A**) Identification of *Escherichia coli* strains by CHROMagar orientation medium; (**B**) Identification of ESBL-producing *E. coli* strain by CHROMagar orientation medium; (**C**) Identification of ESBL-producing *E. coli* strain by double disk synergy test. Original Images of full-length gels are presented in Supplementary Figs. 1, 2, and 3.

The experimental protocol used in this experiment was reviewed and approved by the Animal Experimental Committee of Sichuan Agricultural University and all methods were carried out in accordance with relevant guidelines and regulations. In total, 222 *E. coli* isolates were recovered from 230 nonduplicate cloacal swabs from healthy beef cattle in Sichuan-Chongqing Circle of China. The samples came from 15 beef cattle farms in Yibin and Luzhou. All cloacal swabs were preserved on ice and transported to the laboratory where single *E. coli* isolate was examined per sample. All the *E. coli* strains were isolated and identified by MacConkey agar, Eosin methylene blue agar, and CHROMagar orientation (Fig. 2A) medium^34^. In addition, strains were further confirmed by API 20E system. Confirmed strains of *E. coli* were suspended in 25% glycerol plus tryptic soy broth and stored at − 80 °C.

### Phenotypic screening of ESBL-producers

All *E. coli* strains were subjected to ESBL phenotypic confirmation through the double disk synergy test in line with the guidelines of Clinical Laboratory Standards Institute (CLSI, 2014)^35^, using cefotaxime (30 μg), ceftazidime (30 μg), cefotaxime + clavulanic acid (30/10 μg), and ceftazidime + clavulanic acid (30/10 μg) disks (GE Hangwei Medical Systems Co., Ltd., Beijing, China). A zone of inhibition of ≥ 5 mm was considered positive. For the identification of ESBL producing *E. coli* by CHROMagar orientation medium (Fig. 2B,C), the colony of *E. coli* ESBL strain showed pink to wine red color. *E. coli* ATCC 35,218 (TEM-1 beta lactamase) and *E. coli* ATCC 25,922 were used as positive and negative controls, respectively.

### DNA extraction

Total genomic DNA was extracted from isolates using the TIANamp Bacteria DNA kit (Tiangen Biotech, Beijing, China) according to the manufacturer's instructions. DNA samples were stored at − 20 °C. DNA was amplified by Polymerase Chain Reaction (PCR) amplification. The PCR products were separated by gel electrophoresis in a 1.2% agarose gel stained with GoldView™ (Sangon Biotech, Shanghai, China), visualized under ultraviolet light and photographed using a gel documentation system (Bio-Rad, Hercules, USA).

### Genotypic screening of beta lactam genes

**Figure 3:**
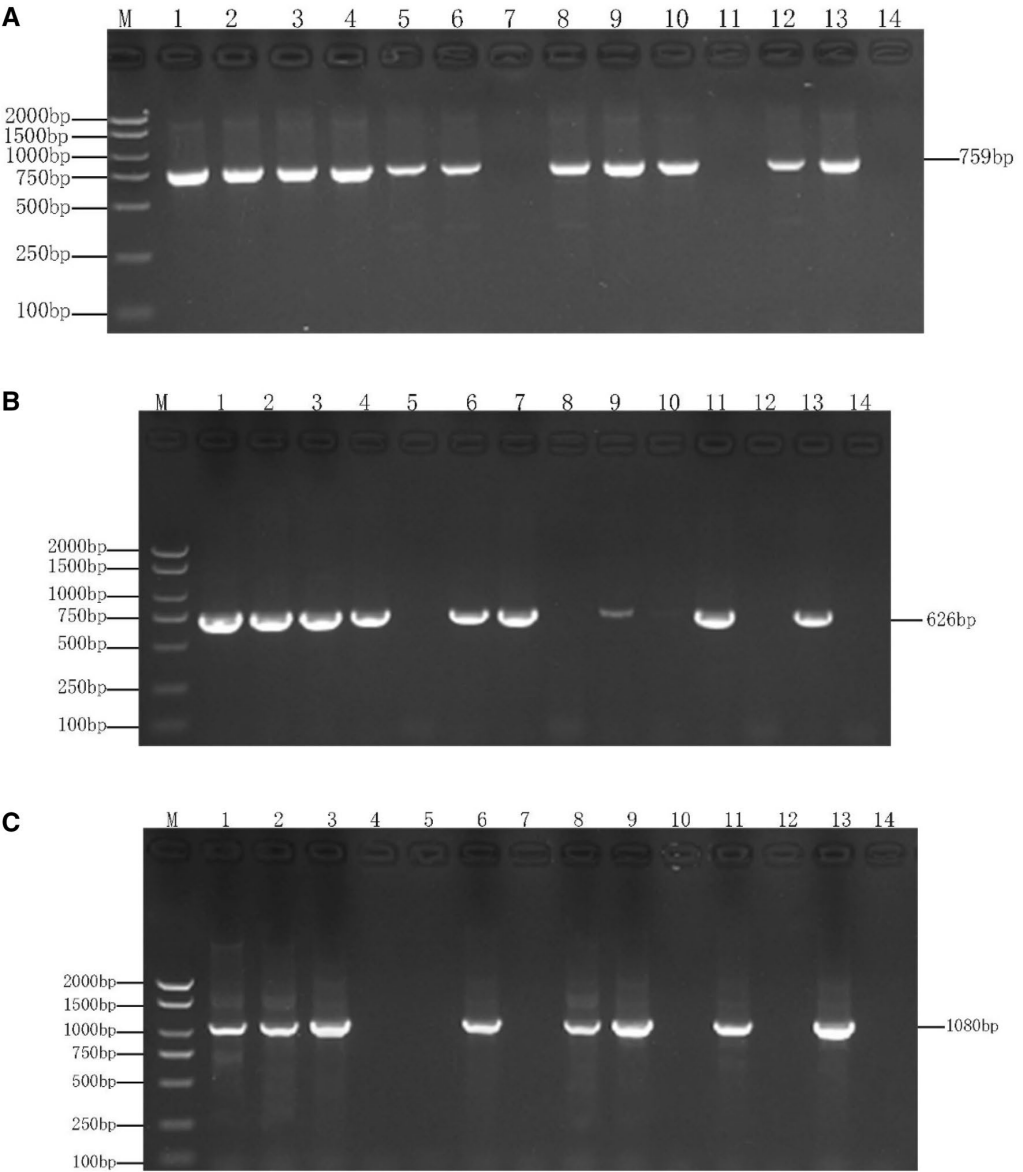
(**A**) Genotypic identification of *bla*_CTX-M_ gene via PCR in *E. coli* strains isolated from beef cattle in china's Sichuan-Chongqing Circle; where, M: 2000 bp DNA Marker, 1: positive controls, 7 and 11: CTX-M negative isolates while others are positive, and 14: negative controls. Original Images of full-length gels are presented in Supplementary Fig. 4. (**B**) Genotypic identification of *bla*_SHV_ gene via PCR in *E. coli* strains isolated from beef cattle in china's Sichuan-Chongqing Circle; where, M: 2000 bp DNA Marker, 1: positive controls, 5, 8, 10, and 12: SHV negative isolates while others are positive, and 14: negative controls. Original Images of full-length gels are presented in Supplementary Fig. 5. (**C**) Genotypic identification of *bla*_TEM_ gene via PCR in *E. coli* strains isolated from beef cattle in china's Sichuan-Chongqing Circle; where, M: 2000 bp DNA Marker, 1: positive controls, 4, 5, 7, 10 and 12: TEM negative isolates while others are positive, and 14: negative controls. Original Images of full-length gels are presented in Supplementary Fig. 6.

Primers and conditions of polymerase chain reaction used in this study are listed in Table 1. The beta lactam genes *bla*_CTX-M_^36^, *bla*_SHV_^37^ and *bla*_TEM_^38^ were amplified with an expected fragment of 759-bp, 626-bp, and 1080-bp, respectively (Fig. 3). All experiments used *E. coli* ATCC 35,218 (TEM positive strain) and *Klebsiella pneumoniae* ATCC 700,603 (SHV and CTX-M positive strain) as the positive control, and sterile water as the negative control. The PCR products were purified and sequenced by Beijing Genomics Institute (BGI), CO., Ltd. (Chengdu, China). The resulting nucleotide sequences were compared with NCBI GenBank and the sequence on http://www.lahey.org/studies/ website for confirmation.

**Table 1:** Primers and conditions of PCR.

Primer	PCR primers (5′ → 3′)	Expected size (bp)	PCR conditions	Ref.
CTX-M-F	ACGCTGTTGTTAGGAAGTG	759 bp	94 °C, 5 min; 35 cycles of 94 °C, 45 s, 58 °C, 45 s, 72 °C, 1 min	^36^
CTX-M-R	TTGAGGCTGGGTGAAGT			
SHV-F	TCGGGCCGCGTAGGCATGAT	626 bp	94 °C, 10 min; 35 cycles of 94 °C, 1 min, 60 °C, 1 min, 72 °C, 1 min	^37^
SHV-R	AGCAGGGCGACAATCCCGCG			
TEM-F	ATAAAATTCTTGAAGACGAAA	1080 bp	94 °C, 5 min; 35 cycles of 94 °C, 1 min, 58 °C, 1 min, 72 °C, 1 min	^38^
TEM-R	GACAGTTACCAATGCTTAATCA

### Phylogenetic grouping

All the beta-lactamase positive *E. coli* strains were allocated to one of the four phylogenetic groups i.e. A, B1, B2 or D by targeting chuA, yjaA, and the DNA fragment TspE4.C2 via triplex PCR assay. As previously described by Clermont, the results were interpreted consequently as recommended^39^.

### Multi-locus sequence typing (MLST) of beta-lactamase producing *E. coli* strains

According to the MLST database guidelines (http://mlst.warwick.ac.uk/mlst/dbs/Ecoli/), seven housekeeping genes (*adk*, *fumC*, *gyrB*, *icdF*, *mdh*, *purE,* and *recA*) were amplified by PCR for MLST analysis following the procedure of Wirth^40^. Two representative isolates were randomly selected from each farm for MLST analysis. Analysis from the seven alleles in the MLST database was further used to determine sequence types (STs) and allele maps.

## Results

### Occurrence of ESBLs among tested isolates

Among the 222 strains of *E. coli* detected by double disk synergy method and CHROMagar orientation medium, 102 strains (45.9%) showed the ESBL phenotype. PCR further detected the occurrence of *bla*_CTX-M_, *bla*_SHV_ and *bla*_TEM_ genes in all ESBL-producing strains. Eighty-eight strains (39.6%) of *E. coli* showed both positive amplified bands and ESBL phenotype. Table 2 shows the frequency distribution of beta lactam genes among *E. coli* isolates from different farms in Yibin and Luzhou regions of Sichuan-Chongqing Circle, China.

**Table 2:** The occurrence of the beta lactam genotypes in different farms of the beef cattle of Sichuan-Chongqing Circle, China (n = 222).

Farm no.	Origin	No. of positive isolates for beta lactam genes						
		CTX-M	TEM	SHV	CTX-M + TEM	CTX-M + SHV	TEM + SHV	CTX-M + TEM + SHV
1	Luzhou (10)				2			
2	Luzhou (15)				2			
3	Luzhou (15)	1						
4	Yibin (15)	3		1	1	1	1	
5	Yibin (15)	3		2	4	3		1
6	Yibin (17)		2	1			1	1
7	Yibin (15 )	3	1					
8	Yibin (15)	1		1				
9	Yibin (15)		1	2		1		1
10	Yibin (15)			1		1		
11	Yibin (15)	3		3		1	2	
12	Yibin (15)	2	3	1	3			
13	Yibin (15)	1		3	4	1	4	
14	Yibin (15)	2		2		2	1	
15	Yibin (15)	2		1	2		1	1
Total (222)		21	7	18	18	10	10	4

The results show that the prevalence of CTX-M and SHV enzymes in the beef cattle of the Sichuan-Chongqing Circle were very high. In addition, the PCR further analysis (Fig. 2) showed that these 88 positive isolates were comprised of 21 *bla*_CTX-M_ strains, 7 *bla*_TEM_ strains, 18 *bla*_SHV_ strains, and 18 strains (containing both *bla*_CTX-M_ + *bla*_TEM_ genes), while 10, 10, and 4 isolates were positive for the combination *bla*_TEM_ + *bla*_SHV_, *bla*_CTX-M_ + *bla*_SHV_, and *bla*_CTX-M_ + *bla*_SHV_ + *bla*_TEM_ genes, respectively.

### Phylogenetic grouping and multilocus sequence typing of *bla*_CTX-M_ producers

**Figure 4:**
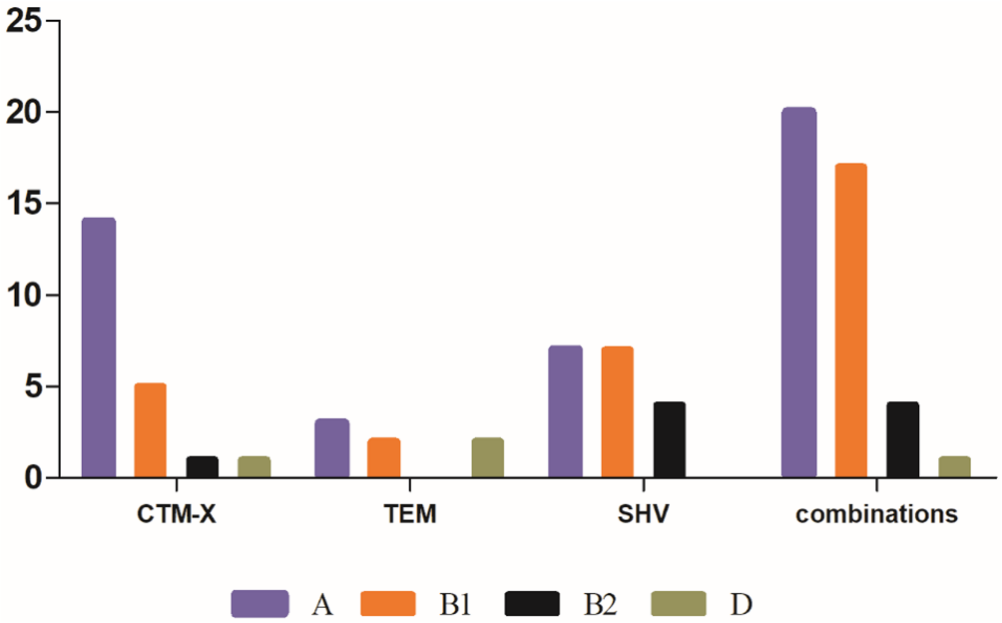
Distribution of beta lactam genes (*bla*_CTX-M_, *bla*_TEM_, *bla*_SHV_) and their combinations into various phylogroups (n = 88).

Using the triplex PCR assay, 88 strains of *E. coli* producing *β-lactamase* were assigned to different phylogenetic groups. The results showed that, majority of the isolates belonged to phylogroup A (n = 44), followed by B1 (n = 30), B2 (n = 9) and D (n = 5). As shown in Fig. 2, CTX-M, or SHV genes was the most prevalent gene detected in phylogroup A or phylogroup B1 in ESBL-producing *E. coli* strains isolated from beef cattle in Sichuan-Chongqing Circle, China (Fig. 4).

Table 3 shows the ST types of representative samples from each farm in the study area and the distribution of each system group. The STs of all tested isolates matched with the MLST database. Obviously, 19 sequence types (ST) were identified from 30 tested isolates. Sequence 398 type (398 Cplex) and sequence 7130 type were the most common, accounting for about 10%. Furthermore, the allelic profiles of 8 (2 isolates each) isolates were similar to ST297, ST48, ST4977 and ST202. However, 2 isolates (CY203 and CY214) were not assigned with an ST type as the housekeeping gene (icd) of these isolates could not be amplified through PCR. Notably, the sequence type 398 and type 7130 isolates produced CTX-M and SHV alone respectively or in combination with other beta lactam genes. In addition, sequence type 398 and type 7130 also belonged to phylogenetic group A and group B2, respectively (Table 3).

**Table 3:** Distribution of ESBL producing *E. coli* isolates into phylogroups, Sequence type (ST) and sequence type complex (STc) identified in the present study (n = 30).

Farm no.	Isolate ID	Phylogroup	Sequence type (ST)	Sequence type complex (STc)
1	CY3	A	10	10Cplex
	CY8	A	398	398Cplex
2	CY12	A	398	398Cplex
	CY15	A	398	398Cplex
3	CY41	A	410	23cplex
	CY53	B1	2144	
4	CY63	B2	297	
	CY66	B1	2179	
5	CY74	A	48	10Cplex
	CY77	A	3856	
6	CY86	B1	602	446Cplex
	CY99	D	1722	
7	CY113	A	4977	206Cplex
	CY114	A	4977	206Cplex
8	CY126	B1	192	
	CY132	B1	539	
9	CY138	B1	1304	
	CY147	B1	2179	
10	CY150	B2	7130	
	CY156	B2	7130	
11	CY164	A	202	
	CY169	A	746	10Cplex
12	CY180	A	202	
	CY186	B2	7130	
13	CY188	D	297	
	CY192	B1	4681	469Cplex
14	CY202	A	48	10Cplex
	CY203	B1	-	
15	CY214	B1	-	
	CY221	A	206	206Cplex

## Discussion

To the best of our knowledge, this is the first comprehensive report to examine the molecular characteristics of ESBL-producing *E. coli* from beef cattle in Sichuan-Chongqing Circle, China. In this study, 45.9% of the isolates were ESBL-phenotypes, which was almost similar to previous reports from South Africa and Germany^41,42^, however, this is higher than the results reported for ESBL-phenotypes in humans and animals, in China. Also, PCR results showed that 39.6% of the isolates expressed *bla*_CTX-M_, *bla*_SHV_ and *bla*_TEM_ 3 genes, of which 21 were *bla*_CTX-M_ strains, 7 *bla*_TEM_ strains, and 18 *bla*_SHV_ strains and 42 strains were positive for a combination of 2 or all. Notably, as previously reported, the *bla*_CTX-M_ group genotype was related to ExPEC *E. coli*, and was one of the main zoonotic risks associated with antibiotic resistance of animal-derived *E. coli*^43^. These isolates were phenotypically resistant to the most commonly used *β-lactam*, and were found to be highly resistant to penicillin derivatives and third-generation cephalosporins which are drugs normally used in China. The main ESBL genotypes tested were *E. coli* sequence type 398 and type 7130. From these findings, we therefore speculate that CTX-M and STs (398) are associated with *E. coli* infections (ExPEC), therefore needs urgent concern as it poses threats to both animal and public health^44^. The spread of antibiotic resistance and toxic properties in food-producing animals has become another challenge for drug resistance control in China, which is not easily restricted compared to the health-care system.

Among the ESBL positive *E. coli*, the results showed that the phylogenetic group A was the most common, followed by B1 and B2 phylogenetic groups. However, there were only 5 positive isolates in phylogenetic group D. It has been previously reported that phylogenetic groups A isolated from animals were more common in tropical regions^45,46^, phylogenetic group B1, B2 and D are said to be associated with ExPEC infections in humans and animals^47,48^. Thus, the distribution of phylogenetic groups may be affected by their geographical region, host species, age, gut morphology, diet, or climate. However, the prevalence of strains belonging to group A in the current study was similar to a previous report from the South Korea^49^. In addition, the high prevalence of phylogenetic group A identified in the appurtenance of the strains isolated from beef cattle in these studies which means there is a high probability of the appearance of superbugs and therefore relevant departments are advised to strictly take action on this public health threat. In addition, the association between phylogenetic group (A) and sequence type (ST398, ST206) was good.

MLST is an accurate molecular typing method, which has been effectively used for typing and establishing clone relationships of isolates. There are more than 7000 strains of *E. coli* in the MLST database. Our results reveal that all the 30 isolates belonged to 19 STs. On the other hand, ST398 and ST7130 accounted for about 20% of the typeable *E. coli* reported in this study. The ST398 *E. coli* isolate that produced CTX-M belonged to line A, with CTX-M genotype; while the ST7130 isolate that produced SHV belonged to line B2, with SHV genotype, however, both combined with other *β-lactam* genes to either produce CTX-M or SHV. ST398 strains are usually associated with infection-related to human. Our results showed that, the ST398 strain was highly detected in the beef cattle, which may be the reason for the success of ST398 as an emerging pathogen^30,44,50^.

Furthermore, our results proved the existence of ST398/ST7130 harboring *bla*_CTX-M_/*bla*_SHV_ in beef cattle in China. Our data also suggested that, the persistence of *bla*_CTX-M_ alleles associated with ST398 infection in humans currently requires additional attention. Therefore, we urgently need to conduct well-designed molecular and epidemiological studies to understand the associated risk factors, reservoirs and transmission dynamics of the *E. coli* ST398/ST7130, which harbors *bla*_CTX-M_/*bla*_SHV_ genes, to help prevent its spread and the health complications caused by ST398/ST7130 worldwide.

In summary, the results of this study showed a high prevalence of CTX-M and SHV enzymes in beef cattle from Sichuan-Chongqing Circle and this finding confirmed their association with *E. coli* infections. Therefore, there is the need for urgent attention as it poses threats to both animal and public health.

## Supplementary Information

Supplementary Figures.

## Data Availability

The datasets used and/or analyzed during the current study are available from the corresponding author on reasonable request.
