# Supplementary material for: High prevalence of blaCTX-M and blaSHV among ESBL producing E. coli isolates from beef cattle in China’s Sichuan-Chongqing Circle
Source: Sci Rep. 2021 Jul 2;11:13725. doi: 10.1038/s41598-021-93201-z (PMC8253751; doi:10.1038/s41598-021-93201-z)
Supplement: Supplementary file 1 — Supplementary Figures. [file 41598_2021_93201_MOESM1_ESM.docx]

**supplementary material (Original Images)**

**1. Supplementary figure 1**


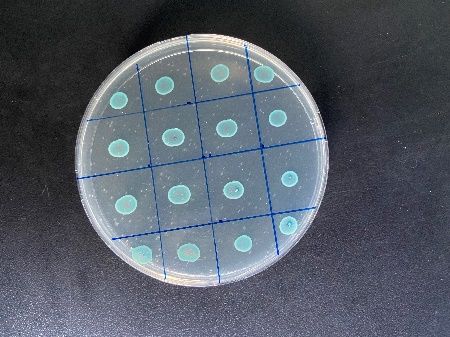


Figure 1. Identification of Escherichia coli strains by CHROMagar orientation medium

**2. Supplementary figure 2**


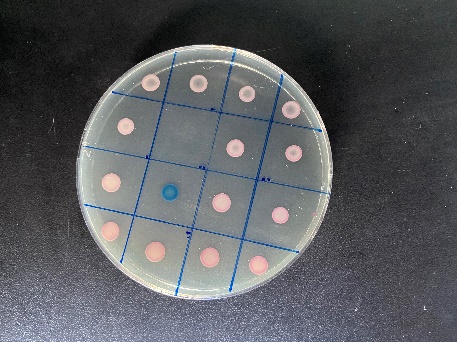


Figure 2. Identification of ESBL-producing E. coli strain by CHROMagar orientation medium

**3. Supplementary figure 3**


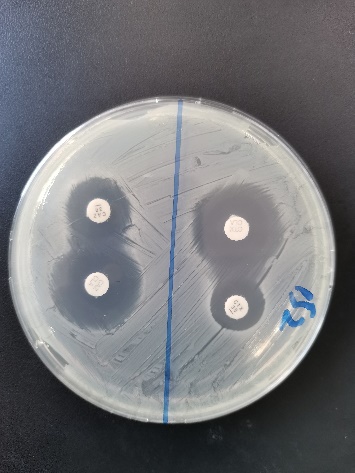


Figure 3. Identification of ESBL-producing E. coli strain by double disk synergy test

**4. Supplementary figure 4**


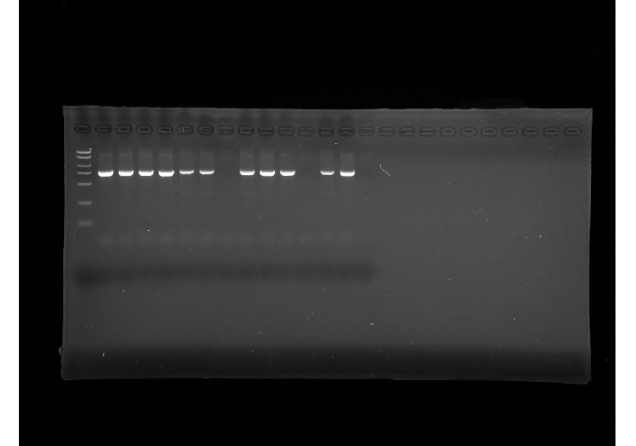


Figure 4. Genotypic identification of *bla*_CTX-M_ gene via PCR in E. coli strains

**5. Supplementary figure 5**


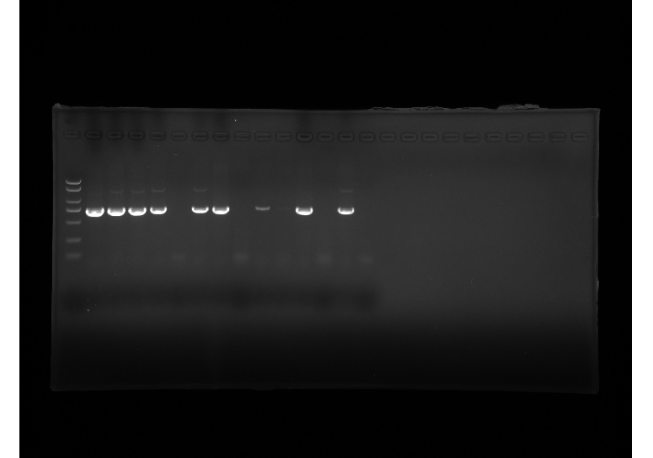


Figure 5. Genotypic identification of *bla*_SHV_ gene via PCR in E. coli strains

**6. Supplementary figure 6**


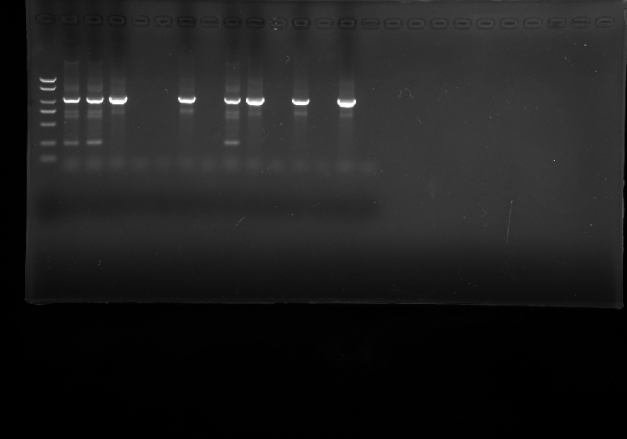


Figure 6. Genotypic identification of *bla*_TEM_ gene via PCR in E. coli strains
